# Sevoflurane Increases Hippocampal Theta Oscillations and Impairs Memory Via TASK-3 Channels

**DOI:** 10.3389/fphar.2021.728300

**Published:** 2021-10-28

**Authors:** Linlin Han, Shuai Zhao, Feng Xu, Yafeng Wang, Ruihui Zhou, Shiqian Huang, Yuanyuan Ding, Daling Deng, Weike Mao, Xiangdong Chen

**Affiliations:** Department of Anesthesiology, Union Hospital, Tongji Medical College, Huazhong University of Science and Technology, Wuhan, China

**Keywords:** local field potentials, memory, sevoflurane, TASK-3 channels, theta rhythms

## Abstract

Sevoflurane can induce memory impairment during clinical anesthesia; however, the underlying mechanisms are largely unknown. TASK-3 channels are one of the potential targets of sevoflurane. Accumulating evidence supports a negative role of intracranial theta rhythms (4–12 Hz) in memory formation. Here, we investigated whether TASK-3 channels contribute to sevoflurane-induced memory impairment by regulating hippocampal theta rhythms. In this study, the memory performance of mice was tested by contextual fear conditioning and inhibitory avoidance experiments. The hippocampal local field potentials (LFPs) were recorded from chronically implanted electrodes located in CA3 region. The results showed that sevoflurane concentration-dependently impaired the memory function of mice, as evidenced by the decreased time mice spent on freezing and reduced latencies for mice to enter the shock compartment. Our electrophysiological results revealed that sevoflurane also enhanced the spectral power of hippocampal LFPs (1–30 Hz), particularly in memory-related theta rhythms (4–12 Hz). These effects were mitigated by viral-mediated knockdown of TASK-3 channels in the hippocampal CA3 region. The knockdown of hippocampal TASK-3 channels significantly reduced the enhancing effect of sevoflurane on hippocampal theta rhythms and alleviated sevoflurane-induced memory impairment. Our data indicate that sevoflurane can increase hippocampal theta oscillations and impair memory function via TASK-3 channels.

## Introduction

Sevoflurane is a commonly used inhaled anesthetic in clinics. Sevoflurane can induce temporary memory impairment to prevent intraoperative awareness during anesthesia. The memory-damaging effect of sevoflurane has been widely confirmed. A functional magnetic resonance imaging (fMRI) study revealed that sevoflurane at subanesthetic concentration (0.25× minimum alveolar concentration) preferentially affected memory-related regions, such as the visual cortices, thalamus and hippocampus (Ramani et al., 2007). Clinical studies have also shown that sevoflurane at approximately 0.25% inhibits human emotional memory (Alkire et al., 2008). Currently, memory is thought to be represented by a set of distributed neurons that are concurrently activated, and brain rhythms are thought to play a key role in memory formation by synchronizing and coordinating the activities of distributed neurons during memory operations (Colgin, 2016). However, the neuroelectrophysiological mechanisms underlying sevoflurane-induced memory impairment, particularly those involving brain rhythms, have yet to be fully elucidated.

The hippocampus is a core brain area responsible for learning and memory (Voss et al., 2017). Hippocampal theta rhythms are synchronized rhythmic oscillations of field potentials at 4–12 Hz that can be produced in the hippocampal CA3 region (Buzsáki, 2002). They could encode the spatial and temporal dimensions that are important for episodic memory, and have been widely demonstrated to play a key role in episodic memory formation (Colgin, 2016; Buzsáki and Tingley, 2018). Evidence suggests that theta rhythms not only are involved in regulating the formation of memory, but also serve as an important electrophysiological index of memory function. Many studies have observed a negative theta effect during memory tasks. A broad decrease in theta power was observed during successful memory encoding in patients with refractory epilepsy (Greenberg et al., 2015). Several other studies confirmed this result, demonstrating decreased theta power during subsequent successful recall tasks (Matsumoto et al., 2013; Long et al., 2014; Fellner et al., 2016). Interestingly, clinical studies have shown that the theta rhythms recorded from the frontal cortex of patients significantly increase during sevoflurane anesthesia (Akeju et al., 2014). These observations prompted us to investigate whether the changes in theta rhythms in electroencephalogram (EEG) are related to sevoflurane-induced memory impairment and to identify the molecular target that mediates the regulation of hippocampal theta rhythms by sevoflurane.

By analyzing the targets of sevoflurane and proteins related to the regulation of theta rhythms, we found that TWIK-related acid-sensitive K^+^ channel 3 (TASK-3) channels may be a potential target for sevoflurane to regulate theta rhythms. TASK-3 channels belong to the two-pore-domain background K^+^ (K2P) family and are abundantly expressed in the hippocampus (Mathie et al., 2020). TASK-3 channels can be activated by clinically relevant concentrations of sevoflurane (Luethy et al., 2017). Notably, the knockout of TASK-3 channels in mice eliminates halothane-increased theta oscillations (Pang et al., 2009). Therefore, we hypothesized that TASK-3 channels may be involved in sevoflurane -induced memory impairment by regulating hippocampal theta rhythms.

## Materials and Methods

### Animals

All experimental procedures conformed to the NIH Guide for the Care and Use of Laboratory Animals and were approved by the Institutional Animal Care and Use Committee of Tongji Medical College. Adult (6–8 week old) male C57BL/6 mice were used in the present study. Four groups of mice were used for behavioral experiments, including fear-conditioning testing (N = 9/group) and inhibitory avoidance testing (N = 9/group). The animals were used for each test independently. For LFPs recording experiments, the animals were subjected to a longitudinal design to observe the effect of sevoflurane on hippocampal LFPs. The mice were housed in groups under a light/dark (12 h/12 h) cycle with *ad libitum* feeding and were tested during the daytime. The experiments and data analysis were carried out in a blinded manner.

### Anesthetic Responses to Sevoflurane

The loss of righting reflex (LORR) was tested to investigate the hypnotic properties of sevoflurane (Tai et al., 2014). To determine the sevoflurane concentration that caused the LORR, mice were individually placed in a plastic chamber. The chamber contained an inlet for gas delivery and an outlet for gas discharge and concentration monitoring. First, 1.0% sevoflurane was vaporized into the chamber, and the concentration of sevoflurane was continuously monitored with an infrared gas monitor (PM8050; Drägerwerk, Germany). After 15 min of equilibration at each concentration, the mice were rolled onto their backs, and the righting reflex was observed for 30 s. A mouse was considered to have experienced the LORR if it failed to turn onto all four feet during the observation period. The sevoflurane concentration was increased by 0.1% until the LORR occurred. At each concentration, the percentage of mice exhibiting the LORR was calculated.

### Behavioral Experiments of Memory

Contextual fear conditioning and inhibitory avoidance experiments were used to evaluate the memory performance of mice (Alkire and Gorski, 2004; Zhong et al., 2014). Mice were acclimated for 3 days prior to the experiment and were given at least 3 h prior to training to habituate to the experimental environment. During the training session, mice were pre-exposed to sevoflurane for 30 min. After that, the mice were quickly transferred into the training chamber, which was filled with the same concentration of sevoflurane. During the testing phase, mice received neither shock nor drugs. Mice in the control group were exposed to air.

#### Fear Conditioning

Training was performed in a fear conditioning chamber (40 cm L × 30 cm W × 26 cm H). After a 3 min exploratory period, mice were individually conditioned with four presentations of a 2-s electric foot shock (1.0 mA), with an interval period of 120 s. After the last foot shock, mice were given an additional 2 min before being returned to their home cages. The next day, mice were individually placed back in the training chamber for 5 min. The freezing behaviors of mice were analyzed with the Xeye software (Zhongshi Technology, China), and their memory performances were evaluated by the percentage of freezing time and number of freezing bouts.

#### Inhibitory Avoidance

The training chamber consisted of a bright (safe) and a dark (shock) compartment that were separated by a door. First, mice freely explored the bright compartment for 3 min (Alkire and Gorski, 2004). After that, the door was opened. If a mouse entered the dark compartment with all four paws, it received a 1.0 mA foot shock until it returned to the bright room. If the mouse did not enter the dark compartment within 100 s, the training was complete; if the mouse entered the dark compartment within 100 s, it was foot shocked again until it returned to the bright compartment and stayed there for more than 100 s. Memory was tested 24 h after training. The latency to avoid darkness and the times of entry into the dark compartment of mice during the observation period (600 s) were recorded.

### Hippocampal Local Field Potential Recording and Analysis

#### Electrode Implantation

Animals were anesthetized with ketamine/xylazine (120/10 mg/kg) and head-fixed into a stereotaxic frame (Rayward, China). Two electrodes were implanted in the hippocampal CA3 regions (AP: −1.7 mm; ML: ± 2.1 mm; DV: −2.1 mm) and a reference electrode was placed over the cerebellum (0.0 mm lateral, 2.0 mm caudal to lambda) (Pang et al., 2009). All electrodes were skull-fixed with dental cement. Recordings were conducted after a 7 days recovery period. The LFP recordings were derived from the potential difference between the recording electrode and the reference electrode. In the present study, only the LFP data recorded from the unilateral (right) hippocampus were analyzed in the present study.

#### Hippocampal Local Field Potentials Recordings

Mice were individually placed into a custom-made 10 L airtight chamber with an inlet for gas delivery and an outlet for gas discharge and concentration monitoring. After the mice adapted to the environment for 5 min, the LFP in the hippocampal CA3 region was recorded in air for 30 min as a control. Following that, sevoflurane and oxygen were continuously input to achieve and maintain the target concentration. After 15 min of equilibrium, the LFPs were recorded for 30 min as a test. Mice behavior during recording was continuously recorded and analyzed by the Xeye software (Zhongshi Technology, China). In the present study, only the data recorded while immobile were primarily analyzed. The LFP data before and after sevoflurane anesthesia were compared. After electrophysiological recording, the locations of the electrodes were observed.

#### Data Analysis

The hippocampal LFP data before and after sevoflurane treatment were bandpass filtered (0.1–50 Hz) and continuously sampled (1 kHz/s) with PowerLab 8/35 (ADInstruments, Australia). The data were then analyzed using LabChart 8.0 (ADInstruments, Australia) and MATLAB (MathWorks R2017a). The raw data were bandpass filtered to extract the signals in the theta frequency band (4–12 Hz). The data were then grouped according to the behavior of the mice, and the filtered data were subdivided into segments of 4,096 points each with 50% overlap, as previously reported (Perouansky et al., 2007; Perouansky et al., 2010). Shorter data were excluded from analysis. All parameters discussed below are the average values of all the segments obtained from animals with the same drug conditions and behaviors (Perouansky et al., 2007; Perouansky et al., 2010). For spectral analysis, the power spectral density (PSD) was generated by LabChart software using Hanning window cosine with 50% window overlapping (Reakkamnuan et al., 2017). The spectrograms were obtained by a modified fast Fourier transform using the pwelch function in MATLAB. Quantitative EEG analysis was performed using custom written MATLAB scripts. The power spectral density and bandpower functions employing FFT in MATLAB were then used to obtain the spectral band power in each frequency range. The power was then transformed to a decibel scale through a logarithmic transformation [Power(dB)=10⁡log⁡10(Power(μV))] .

### Stereotaxic Virus Injection

Adeno-associated virus (AAV, serotype 9) that expressing nontarget (NT) shRNA or TASK-3 shRNA was prepared by Vigene Biosciences as previously reported (Wu et al., 2012; Liu et al., 2018). The sequence of TASK-3 shRNA is 5′-GCT​GGT​GTC​CAG​TGG​AAA​TTC-3′ (Choi et al., 2018). The shRNA sequences were cloned into the pAAV-U6-GFP vector under the control of the U6 promoter. The tri-plasmids system was then used to generate the recombinant AAV expression vector in 293T cells. The virions were then purified after 72 h of cell culture. NT shRNA was used as a control. Animals were anesthetized with ketamine/xylazine (120/10 mg/kg) and head-fixed into a stereotaxic frame (Rayward, China). Mice were administered a bilateral injection of 0.5 μl of viral solution into the CA3 regions via a glass micropipette. The injection speed was controlled at 0.1 μl/min by using a microsyringe pump (Rayward, China). To prevent reflux, the pipette remained *in situ* for 5 min after injection before being withdrawn stepwise (0.5 mm/step, 3 min/interval). The scalp was then closed, and the animals were housed for 1 month before subsequent experiments. Mice were given an additional 3 days to acclimate to the environment and investigators before behavioral experiments. In the present study, the air control groups were run as separate batches.

### Histology and Image Analysis

The animals were transcardially perfused with physiological saline and 4% paraformaldehyde (PFA) successively. The brain was then removed and placed in PFA for an additional 24 h. Subsequently, each brain was sectioned into 4 μm-thick sections and stained overnight at 4°C with a TASK-3 antibody (1:400; NBP1-46493, Novus). After that, the tissues were labeled for 1 h with AlexaFluor 488-conjugated secondary antibodies (ab150129; Abcam) The nuclei were stained with 4′,6′-diamidino-2-phenylindole (DAPI). TASK-3-positive cells were manually counted in the CA3 region of the dorsal hippocampus under a fluorescence microscope (Olympus) at 100× magnification. A rectangular area (CA3: 540 × 720 μm) was drawn to limit cell counting in the CA3 region (Oishi et al., 2019). For each mouse, three nonadjacent brain sections that contained the area of interest were analyzed.

### Whole-Cell Recording

TASK-3 cDNA and green fluorescent protein (GFP) were cotransfected into human embryonic kidney (HEK) 293 cells with Lipofectamine 2000 (Invitrogen). Whole-cell recordings were performed at room temperature in a bath solution containing the following components (in mM): 130 NaCl, 3 KCl, 2 MgCl_2_, 2 CaCl_2_, 10 HEPES, and 10 glucose, with the pH adjusted using HCl and NaOH (Lazarenko et al., 2010; Du et al., 2011). Recording pipettes had a DC resistance of 3–5 MΩ when filled with the following pipette solution (in mM): 120 KCH_3_SO_3_, 4 NaCl, 1 MgCl_2_, 0.5 CaCl_2_, 10 HEPES, 10 EGTA, 3 MgATP, and 0.3 GTP-Tris, pH 7.2 (Lazarenko et al., 2010; Du et al., 2011). Cells were held at −60 mV before undergoing a series of ramp voltage commands (0.2 V/s, ranging from −130 to 20 mV).

### Statistical Analysis

GraphPad Prism 8 (GraphPad Software, United States) was used for the calculation and analysis of all experimental values. The 50% effective concentration (EC_50_) for the LORR was obtained from dose-response data that had been curve-fitted in Prism by nonlinear regression. The 95% CI of the EC_50_ was also calculated using Prism. Data comparisons were analyzed by one-way ANOVA followed by Bonferroni’s *post-hoc* tests, unpaired t-tests or paired t-tests. Values are presented as the means ± SEM, and differences were considered significant when *p* < 0.05.

## Results

### Sevoflurane Significantly Impairs the Memory Functions of Mice

**FIGURE 1:**
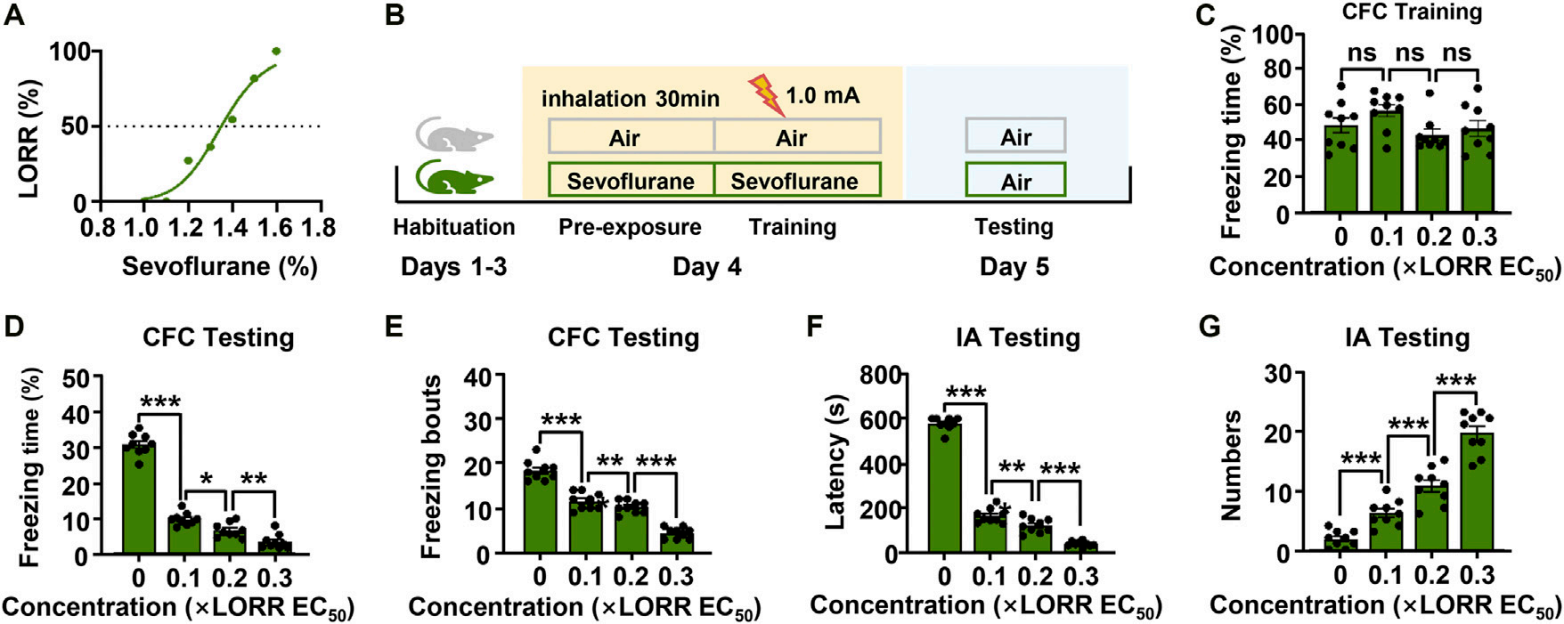
Subanesthetic concentrations of sevoflurane impair fear-conditioned memory in mice. **(A)** The dose-response curve to sevoflurane for mice. The data were curve-fitted using nonlinear regression. **(B)** Diagram of the classical fear conditioning experiment. **(C)** Learning evaluation of mice during the training session of the contextual fear conditioning (CFC) test. *N* = 9/group. **(D, E)** Bar graphs showing the percentage of freezing time and number of freezing bouts for mice in different groups when they were re-exposed to the conditioning context. **(F, G)** In the inhibitory avoidance (IA) experiment, the time taken before the trained mice again crossed into the shock compartment and the times for mice to enter the shock compartment during the test period. *N* = 9/group. Values are shown as the mean ± SEM; one-way analysis of variance (ANOA) with Bonferroni post hoc test. *p < 0.05, **p < 0.01, ***p < 0.001.

The EC_50_ for the LORR of sevoflurane was 1.4% (95% CI, 1.3–1.4%) (Figure 1A). We then investigated the effect of different concentrations of sevoflurane (0.1, 0.2, 0.3 × LORR EC_50_) on memory by using a contextual fear conditioning experiment (Figure 1B). The freezing percentages of mice in the four groups were comparable during the training session, indicating that all the mice learned the task information (Figure 1C). During the testing session, the percentage of freezing time and number of freezing bouts of sevoflurane-anesthetized mice were significantly lower than those of control mice. The freezing behaviors in sevoflurane-anesthetized mice were further reduced with increasing sevoflurane concentration, and the differences were significant when compared to the previous concentration (Figures 1D,E).

To confirm the results above, we performed a second memory behavior test in mice. In the inhibitory avoidance experiment, sevoflurane also had a significant effect on the memory performance of mice (Figures 1F,G). Mice in the control group showed robust memory, with latencies of 577.8 ± 9.6 s and 1.8 ± 0.5 entries into the dark compartment. However, sevoflurane-anesthetized (0.1× LORR EC_50_) mice exhibited clear memory impairment, with decreased latencies (167.3 ± 10.8 s) and increased entries into the dark compartment (6.1 ± 0.7 entries). In addition, as sevoflurane concentration increased, the memory functions of mice were further impaired [0.2× LORR EC_50_ (124.3 ± 10.6 s, 10.7 ± 1.0 entries), 0.3 × LORR EC_50_ (42.7 ± 4.1 s, 19.6 ± 1.1 entries)] (Purdon et al., 2015). These data suggest that sevoflurane at subanesthetic concentrations impaired the memory functions of mice, and that this effect is enhanced in a dose-dependent manner. Additionally, these results indicated that 0.2 × LORR EC_50_ sevoflurane significantly reduced the memory performance of mice. Thus, sevoflurane at 0.2 × LORR EC_50_ was chosen for the subsequent experiments.

### Sevoflurane (0.2 × LORR EC_50_) Increases the Power of Hippocampal Theta Rhythms

**FIGURE 2:**
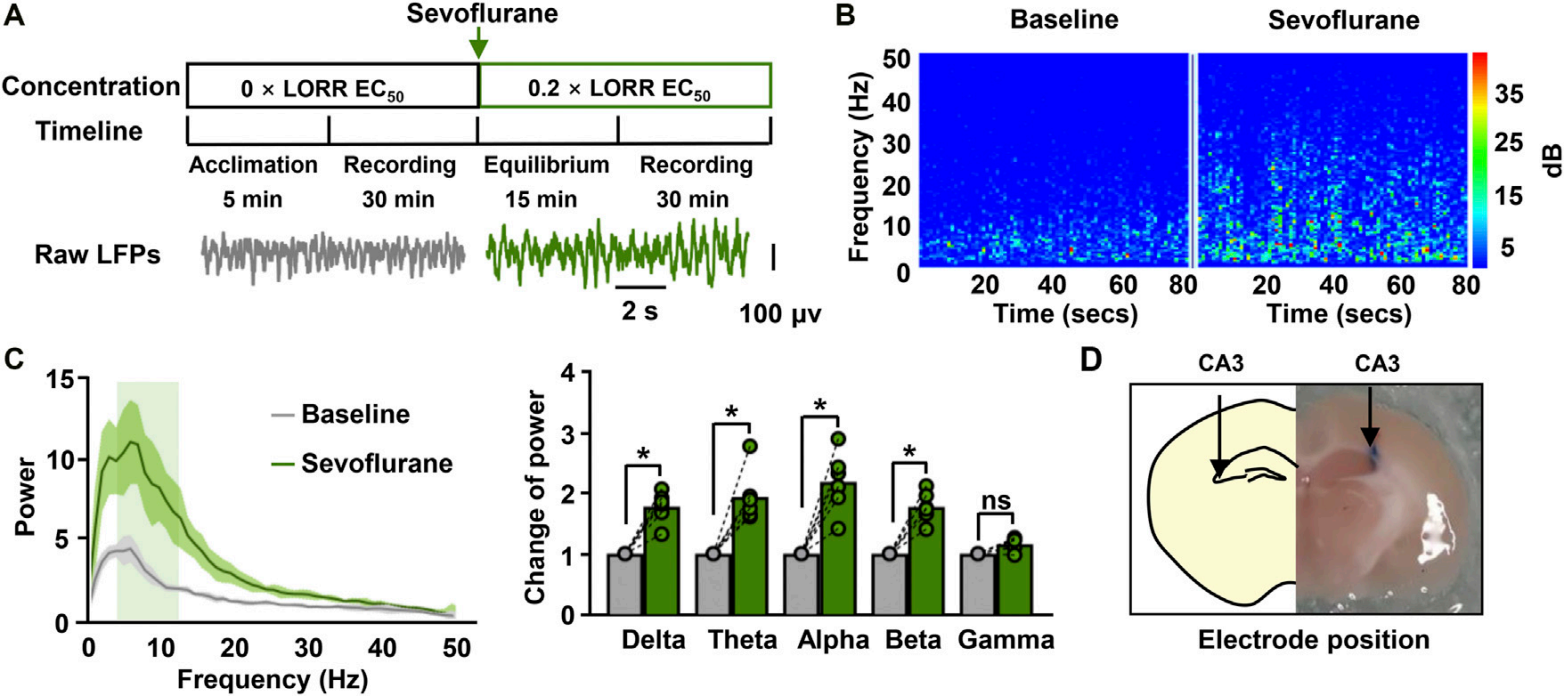
Hippocampal Local Field Potential (LFPs) for sevoflurane-anesthetized mice. **(A)** The timeline for LFP recordings **(upper)** and the raw traces of hippocampal LFPs in a representative mouse before and during sevoflurane anesthesia **(bottom)**. The data were digital-filtered to 1–50 Hz. **(B)** Representative hippocampal spectrograms from sevoflurane-anesthetized mice selected for 80 s. The spectrograms exhibit the frequency content of signals changing with time. The *Y*-axis indicates the frequency, and the *X*-axis indicates the time. **(C)** Representative power spectrum **(left)** of the LFPs recorded during immobility for mice anesthetized with sevoflurane. *Solid lines* represent mean values, and *shaded regions* represent 95% CIs. The bar graphs **(right)** show the changes in total power in each frequency band compared to the base value. *N* = 6/group. **(D)** The location of the electrode implanted in the CA3 region of the hippocampus. Values are shown as the mean ± SEM; paired t-tests. *p < 0.05.

We subsequently characterized the effect of sevoflurane on neural activities *in vivo* by recording hippocampal LFPs in mice. Sevoflurane increased the amplitude of hippocampal LFPs in comparison to the baseline (Figure 2A). This increase was also be found in the spectrogram observed during sevoflurane anesthesia (Figure 2B), which was characterized by higher power across the low-frequency band (1–30 Hz) ranges. The changes in the power spectrum were statistically confirmed. Sevoflurane enhanced power across most frequencies in the range of 1–30 Hz (delta rhythm: 1.8-fold; theta rhythm: 1.9-fold; alpha rhythm: 2.2-fold; beta rhythm: 1.8-fold) (Figure 2C). These data indicate that sevoflurane had strong effects on neuronal activity at memory-damaging concentrations. After electrophysiological recording, the locations of the electrodes were observed (Figure 2D).

**FIGURE 3:**
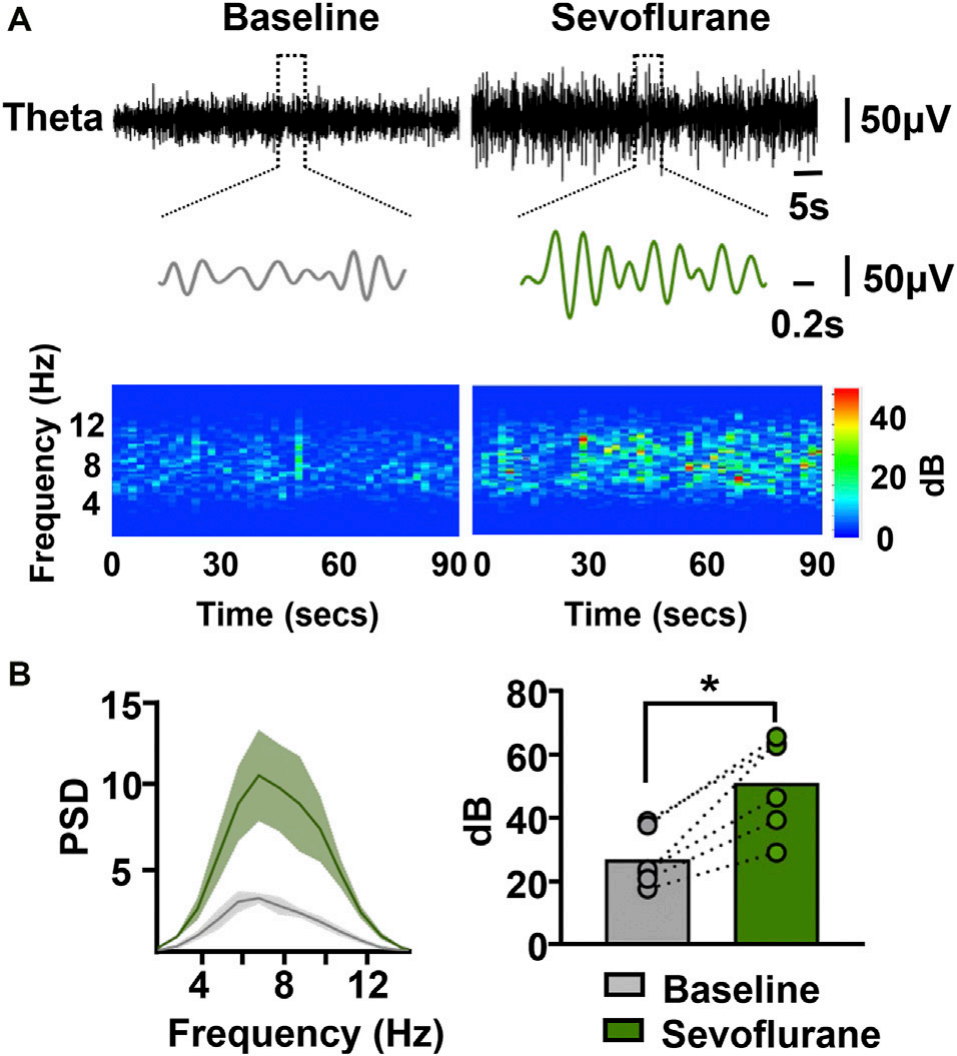
Sevoflurane at memory-damaging concentrations enhances hippocampal theta oscillations in mice. **(A)** Examples of theta waves and representative spectrograms recorded at immobile status prior to and during sevoflurane anesthesia. The data were digital-filtered to 4–12 Hz. **(B)** Representative power spectrum in the theta frequency band **(left)** obtained from a sevoflurane-anesthetized mouse. *Solid lines* represent mean values, and *shaded regions* represent 95% CIs. Sevoflurane significantly increased the power in the theta band **(right)**. *N*=6/group. Values are shown as the mean ± SEM; paired *t*-tests. *p < 0.05.

Hippocampal theta rhythms have been shown to play an important role in episodic memory. We next investigated the effect of sevoflurane on theta rhythms, hypothesizing that memory-related theta rhythms may correlate with sevoflurane-induced memory impairment. Sevoflurane clearly increased the amplitude of theta waves (Figure 3A). The spectrogram was characterized by higher theta power during sevoflurane anesthesia than at baseline. This was further demonstrated by the following data (Figure 3B). A higher peak in the spectral density at the theta frequency was observed during sevoflurane anesthesia. In addition, sevoflurane markedly increased the total power in the theta band from 27.6 to 51.8 dB. These results indicate that the sevoflurane-induced memory impairment is accompanied by an increase in the total power of hippocampal theta rhythms.

### Knockdown of Hippocampal TASK-3 Channels Eliminates Sevoflurane-Enhanced Theta Oscillations

**FIGURE 4:**
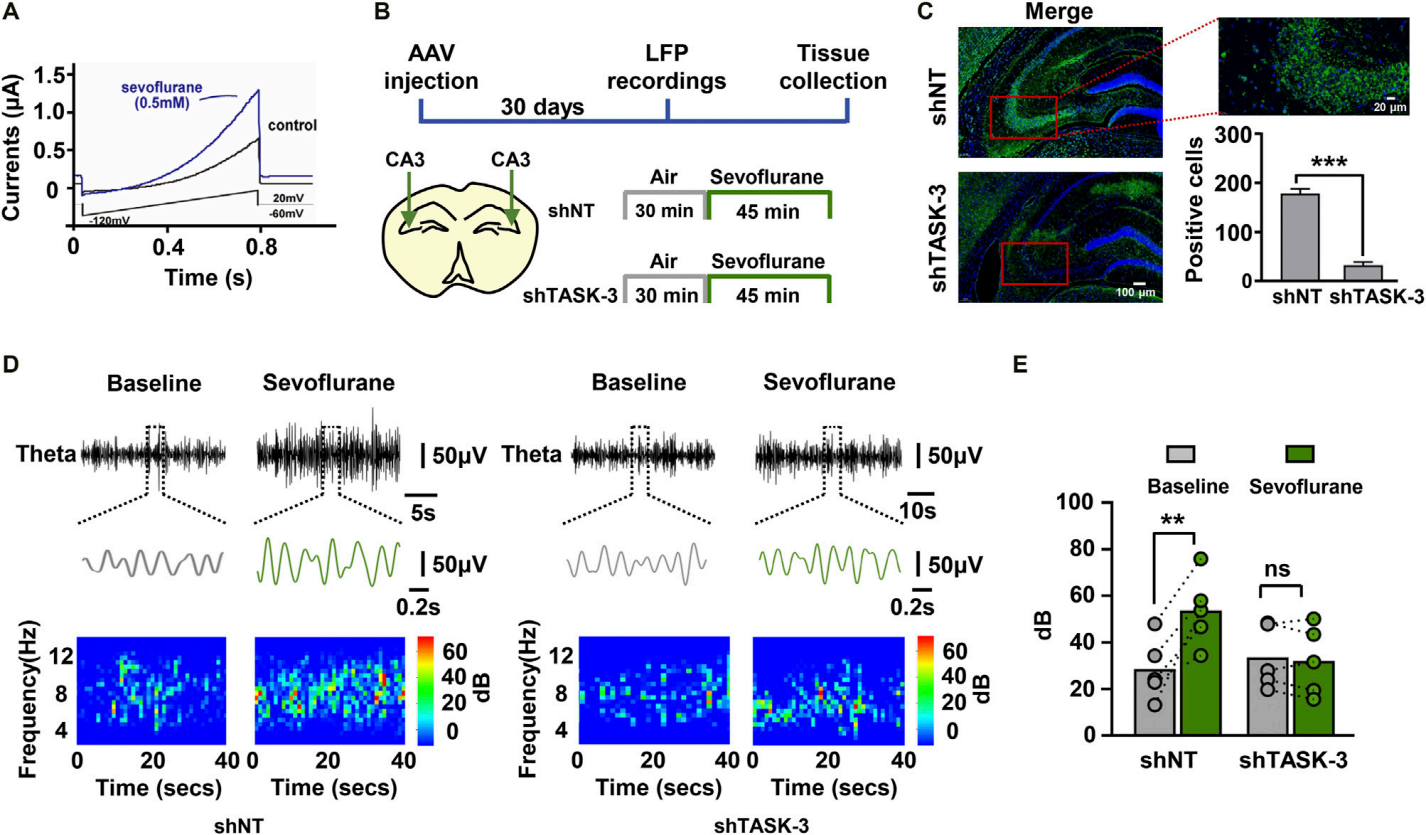
TASK-3 channels mediate the enhancing effect of sevoflurane on theta oscillations. **(A)** Representative time course of whole-cell current during sevoflurane (0.5 mM) application. The TASK-3 construct was transfected into HEK293 cells and ramp voltage commands were used to elicit the TASK-3 current. (**B**) Timeline of experiment. *In vivo* electrophysiological recording was conducted after stable expression of adeno-associated virus (AAV) in mice. **(C)** Representative immunofluorescence images of TASK-3 channels (green) in the hippocampus of both control and TASK-3 knockdown mice. Scale bar, 100 μm in left images. Scale bar, 20 μm in right image. Quantification of TASK-3 antibody-labeled cells in the hippocampal CA3 region from mice with both phenotypes. **(D)** Examples of theta waves and representative spectrograms recorded at immobile status during sevoflurane anesthesia from control **(left)** and TASK-3 knockdown**(right)** mice. **(E)** The total power of theta rhythms from control and TASK-3 knockdown mice before and during sevoflurane anesthesia. *N* = 5/group. Values are shown as the mean ± SEM. Unpaired t-tests shown in (**C**); paired t-tests shown in (**E**). **p < 0.01, ***p < 0.001.

To further elucidate the molecular mechanisms by which sevoflurane regulates theta oscillations, we analyzed many proteins that are sevoflurane-sensitive and can regulate theta rhythm. We identified TASK-3 channels as a potential target. First, we investigated the effects of sevoflurane on the function of TASK-3 channels. The amnestic concentration of sevoflurane was shown to be 0.5 mM (Ishizeki et al., 2008). We observed that TASK-3 currents in transfected HEK293 cells were strongly activated by sevoflurane (Figure 4A. This result is consistent with previous studies showing several inhaled anesthetics, including isoflurane, desflurane and halothane, can activate TASK-3 channels (Luethy et al., 2017). To investigate the potential roles of TASK-3 channels in the regulation of sevoflurane (0.2 × LORR EC_50_) on theta oscillations, we used shRNA to downregulate the expression of TASK-3 channels in the hippocampal CA3 region. One month later, the hippocampal LFPs of the mice were recorded (Figure 4B). Immunofluorescence staining was used to visualize the expression of TASK-3 channels in intact hippocampus, and the knockdown of TASK-3 channels in transduced neurons was detected as a decrease in the number of antibody-labeled neurons when compared with the control group (Figure 4C). The representative theta traces and spectrograms showed that sevoflurane could increase both the amplitude and power within the theta band in control mice but not in TASK-3 knockdown mice (Figure 4D). Correspondingly, sevoflurane failed to increase the total power of the theta band in TASK-3 knockdown mice (Figure 4E). In addition, we also investigated the potential roles of TASK-3 channels in the regulation of other frequency bands by sevoflurane. Our results indicated that the knockdown of hippocampal TASK-3 channels had no effect on sevoflurane-enhanced delta, alpha or beta oscillations (Supplementary Figures 1A–C).

### Knockdown of Hippocampal TASK-3 Channels Alleviates Sevoflurane-Induced Memory Impairment

**FIGURE 5:**
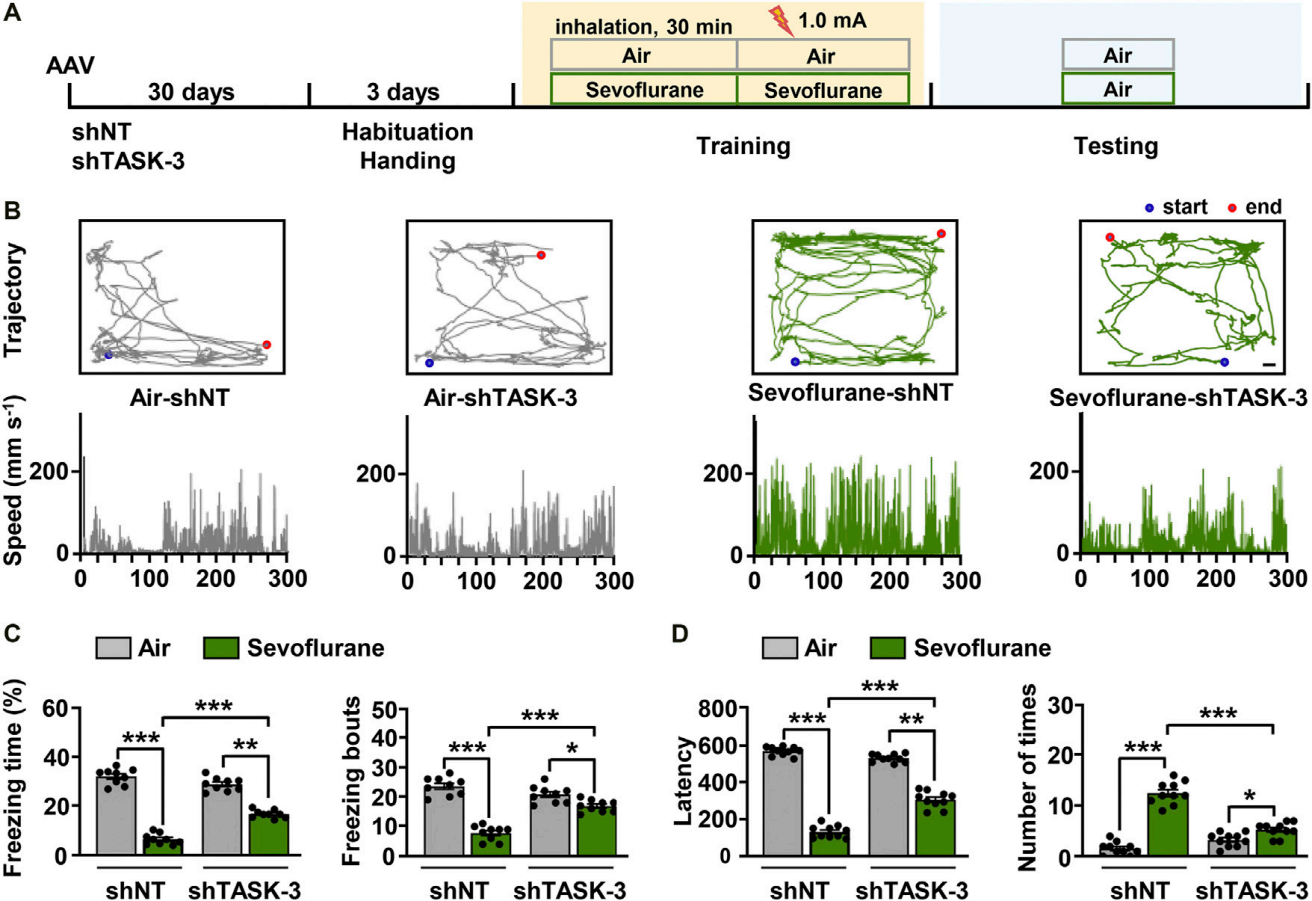
TASK-3 channels mediate sevoflurane-induced memory impairment. **(A)** Timeline of experiment. After adeno-associated virus (AAV) injection for 1 month, mice were fear-conditioned to test their memory performance. **(B)** Locomotor trajectory and corresponding moving speeds of representative mice in four different experimental groups throughout an entire (5 min) testing session. Scale bar, 4.17 cm. **(C)** Bar graphs show the percentage of freezing time and number of freezing bouts for the control and TASK-3 knockdown mice when re-exposed to the conditioning context. *N* = 9/group. **(D)** The time taken before the trained mice again crossed into the shock compartment **(left)** and the number of times mice entered the shock compartment **(right)**. *N* = 10/group. Values are shown as the mean ± SEM. *p < 0.05, **p < 0.01, ***p < 0.001.

Because sevoflurane was found to regulate theta oscillations via hippocampal TASK-3 channels (Figures 4C,D), we investigated whether the observed changes in theta activity were related to the memory performance of mice. Mice injected with TASK-3 shRNA or NT shRNA in the CA3 region were subjected to fear conditioning training under either sevoflurane (0.2 × LORR EC_50_) anesthesia or air, and their freezing responses were tested the next day (Figure 5A). The results showed that there were no detectable differences in movement trajectories and speeds between mice with and without TASK-3 knockdown in the air group. However, in the sevoflurane group, TASK-3 knockdown mice spent a lower percentage of time exploring and did so at slower speeds than NT shRNA-treated mice (Figure 5B). Correspondingly, the knockdown of TASK-3 channels had no effect on the memory performance of air-exposed mice (29.0 *±* 0.3% *v*s. 32.2 ± 0.3%, 20.8 ± 0.3 *vs.* 23.6 ± 0.3, *p* > 0.05) but was shown to attenuate sevoflurane-induced memory impairment, as indicated by a higher percentage of freezing time and number of freezing bouts than in NT shRNA-treated mice (17.0 ± 0.2% *vs.* 6.8 ± 0.3%, 16.9 ± 0.2 *vs.* 7.8 ± 0.3, *p* < 0.001) (Figure 5C). Notably, the fear responses of TASK-3 knockdown mice were still different between the air and sevoflurane groups, implying that the knockdown of TASK-3 channels could only inhibit rather than block the memory impairment effects of sevoflurane.

The above results were further confirmed using an inhibitory avoidance experiment (Figure 5D). In the air group, mice with and without TASK-3 knockdown showed comparable latencies of avoiding darkness (567.3 ± 7.5 s *vs.* 529.0 ± 6.8 s, *p* > 0.05) and entries into dark room (1.6 ± 0.4 entries *vs.* 3.3 ± 0.4 entries, *p* > 0.05). However, in the sevoflurane group, shTASK-3-treated mice had longer latencies of avoiding darkness and fewer entries into the dark room than those observed in the NT shRNA-treated mice (305.3 ± 13.5 s *vs.* 131.4 ± 9.9 s, 5.3 ± 0.5 entries *vs.* 12.6 ± 0.8 entries, *p* < 0.001). In addition, we observed that the knockdown of TASK-3 channels only inhibits rather than blocks the effects of sevoflurane. These results indicate that the knockdown of TASK-3 channels is sufficient to alleviate sevoflurane-induced impairment of fear-conditioned memory in mice.

## Discussion

The present study aimed to investigate the mechanisms of memory impairment caused by sevoflurane during clinical anesthesia. Our findings reveal that sevoflurane increases hippocampal theta oscillations via TASK-3 channels. Additionally, our results indicate a previously unreported role for TASK-3 channels, in the mechanism underlying sevoflurane-induced memory impairment. It has been reported that theta oscillations are closely related to memory function. Therefore, our results suggest that TASK-3 channels may contribute to sevoflurane-induced memory impairment by enhancing hippocampal theta oscillations.

### Experimental Paradigms

In the present study, we chose contextual fear conditioning and inhibitory avoidance paradigms to investigate the effects of sevoflurane on memory for the following reasons. First, the harmful electric shocks used in animal training can better simulate the traumatic experience of surgical patients. Second, these two behavioral methods were able to evaluate hippocampus-dependent memory (Izquierdo et al., 2016), which have been shown to be more sensitive to inhaled anesthetics than hippocampus-independent memory (Dutton et al., 2001). Third, both contextual fear conditioning and inhibitory avoidance paradigms are well-established behavioral paradigms for testing fear memory and have been widely used to assess the effects of anesthetics on memory (Dutton et al., 2001; Alkire and Gorski, 2004; Knox, 2016). Additionally, it is difficult to maintain a constant concentration of sevoflurane during the training phase of some behavioral methods, such as the Morris water maze.

### Theta Rhythms and Memory Function

In the present study, we observed that sevoflurane anesthesia (0.2 × LORR EC_50_) could significantly increase the power across the low-frequency (1–30 Hz) band ranges. This result is consistent with previous studies (Akeju et al., 2014), which also found increased electroencephalogram power across a range of frequencies (0.4–40 Hz). We focused on the role of theta rhythm in sevoflurane-induced memory impairment for the following reasons. First, previous studies have shown that anesthetic-induced changes in theta rhythm are related to memory function (Perouansky et al., 2010). Second, theta oscillations are widely reported to be correlated with memory function during the awake state. Anesthetic-induced theta oscillations are likely to be distinct from those seen during exploration. We aimed to investigate whether the changes in theta rhythm induced by sevoflurane could also modulate memory function.

One of the principal findings of the present study is that the changes in theta power were negatively correlated with the memory function of mice under sevoflurane anesthesia. We examined hippocampal LFPs and observed that sevoflurane at memory-damaging concentrations could cause a clear increase in theta power. The discovered relationships between theta spectral power and memory function are similar to those found in previous studies of individuals with intracranial recordings, showing that successful memory encoding correlates with decreases in some or all of the theta activity (Sederberg et al., 2007; Burke et al., 2013; Long et al., 2014; Greenberg et al., 2015). These results seem surprising, given that some evidence from noninvasive scalp EEGs have demonstrated that theta activity promotes successful memory formation (Addante et al., 2011). The reasons for this discrepancy remain unknown. Nora and colleagues attributed the disparities in these results to different spatial resolution scales between the two measuring methods (Herweg et al., 2020). Although neocortical theta activity originates from hippocampal projections, scalp EEG measurements are less capable of precisely localizing the source of neural oscillations. More importantly, the spectral power in scalp EEGs is a linear combination of interelectrode synchrony and LFP power. Thus, in the case of increased synchrony, it is possible to detect positively modulated spectral power, even with a negative LFP modulation (Musall et al., 2014; Herweg et al., 2020). In addition to the recording methods, the experimental and analytical methods, such as the activity state of the animals and the methods and criteria for testing successful memory performance, are also correlated with the observed relationship between theta power and memory function (Herweg et al., 2020).

How do the changes in theta activity affect memory formation? It has been speculated that the decreased theta power observed during memory formation reflects a decrease in neuronal correlation between the cortex and the medial temporal lobe, thereby increasing the amount of information that can be stored throughout the network (Greenberg et al., 2015). Studies in humans using depth electrodes showed that successful memory encoding and retrieval are associated with increased theta connectivity within the medial temporal lobe but are accompanied by a decrease in theta spectral power (Solomon et al., 2019). Thus, another possibility is that the increased theta power during sevoflurane anesthesia is due to decreased functional connectivity among medial temporal lobe subregions, resulting in memory impairment. The role of phase-synchronous theta oscillations in memory formation has also attracted much attention. However, the strict relationship between spectral power and phase synchrony has rarely been verified. In the context of the observed decrease in theta power during memory formation, both increased and decreased synchronous theta phases were observed (Burke et al., 2013). Thus, whether and how synchronous theta activities contribute to the memory process remains unknown.

### TASK-3 Channels Mediate the Regulation of Sevoflurane on Memory and Theta Oscillations

Several studies have explored the mechanisms underlying anesthetic-induced memory impairment. GABA receptors with specific subunits have been found to mediate the memory-damaging effects of etomidate, isoflurane and propofol (Solt and Forman, 2007; Garcia et al., 2010). Some studies have highlighted the role of ionic glutamate receptors in rapid excitatory synaptic transmission, synaptic plasticity and cognitive function (Wang and Orser, 2011). Here, we found that TASK-3 channels can mediate sevoflurane-induced memory impairment, and that this may involve theta oscillations.

Numerous studies have shown that anesthetics cause different EEG patterns by interacting with specific molecular targets. Low doses of ketamine induced beta-gamma oscillations primarily by acting on NMDA receptors (Purdon et al., 2015). Dexmedetomidine primarily binds to presynaptic α2 adrenergic receptors and produces EEG patterns that are characterized by slow delta and spindles (Purdon et al., 2015). The EEG dynamics of sevoflurane and propofol anesthesia were similar, consistent with their common effects on GABA receptors (Hemmings et al., 2005). It has been suggested that sevoflurane, similar to propofol, may also potentiate postsynaptic GABA_A_ receptors at the thalamic reticular nucleus, reducing exogenous inputs to the neocortex and causing a state of thalamocortical synchrony, which favors the appearance of alpha oscillations (Bai et al., 1999; Hemmings et al., 2005). In the present study, we found that, sevoflurane at memory-damaging concentrations can also enhance hippocampal theta oscillations and that this effect was mediated by TASK-3 channels. This is consistent with the activation effect of sevoflurane on TASK-3 channels. Both our study and previous studies have found that sevoflurane can activate TASK-3 channels (Luethy et al., 2017). A previous study, which found a loss of the halothane-induced peak in the theta frequency in TASK-3 knockout mice, supports our results (Pang et al., 2009). The findings of a recently published study may provide a reasonable explanation for these phenomena in terms of the relationship between the membrane potential dynamics and theta oscillations. Malezieux and others showed that the theta rhythms are characterized by the hyperpolarization of most CA3 pyramidal cells (Malezieux et al., 2020). TASK-3 channels are responsible for maintaining cellular resting membrane potential, and their activation by various volatile anesthetics increases the outflow of K^+^, resulting in the hyperpolarization of the cell membrane potential (Putzke et al., 2007). Therefore, TASK-3 channels most likely regulate the magnitude of the recorded field potentials by affecting transmembrane currents.

The present experiments were conducted on various groups of animals that were given anesthetics while either measuring hippocampal oscillations or performing behavioral experiments. This is a limitation of our study. First, we cannot artificially modulate hippocampal theta rhythms *in vivo* to rescue the memory impairment caused by sevoflurane; thus, the causality between hippocampal theta rhythm modulation and sevoflurane-induced memory impairment has not been established. A recently published study provides compelling evidence that theta oscillations within and between the amygdala and medial prefrontal cortex constitute a general mechanism of fear learning across species (Chen et al., 2021). More research should be conducted to better understand the relationship between theta oscillations and memory function during anesthesia.

## Conclusion

In conclusion, our study showed that sevoflurane increases hippocampal theta oscillations and impairs memory *via* TASK-3 channels. These findings may deepen the understanding of the molecular mechanisms of general anesthetic action.

## Data Availability

The raw data supporting the conclusion of this article will be made available by the authors, without undue reservation.
