# Supplementary material for: Sevoflurane Increases Hippocampal Theta Oscillations and Impairs Memory Via TASK-3 Channels
Source: Front Pharmacol. 2021 Oct 28;12:728300. doi: 10.3389/fphar.2021.728300 (PMC8581481; doi:10.3389/fphar.2021.728300)
Supplement: Supplementary file 1 [file Table1.DOCX]

# Supplementary Figures and Tables

## Supplementary Figures

##
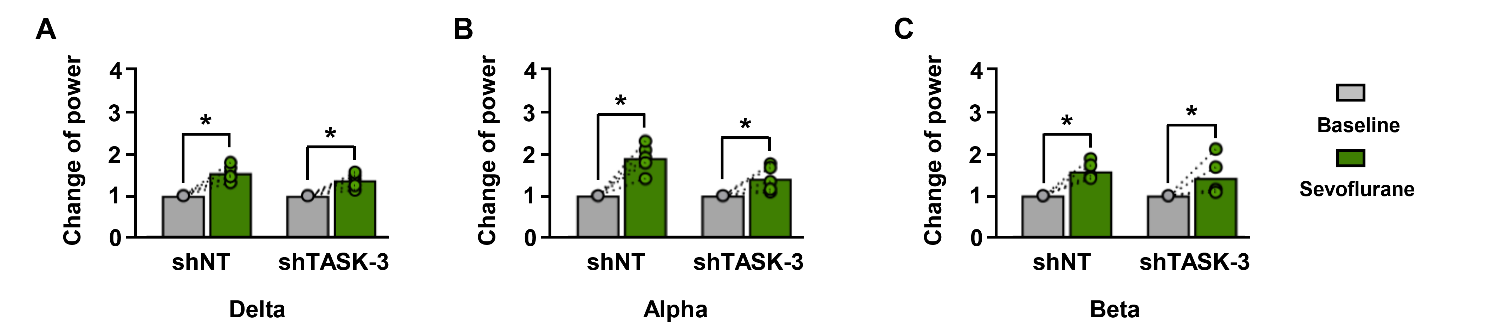


**Supplementary Figure 1.** The total power of delta (A), alpha (B) and beta (C) rhythms from control and TASK-3 knockdown mice before and during sevoflurane anesthesia. N = 5/group. **P* < 0.05.
